# The Missing Link in Correlates of Protective Tuberculosis Immunity: Recognizing the Infected Cell

**DOI:** 10.3389/fimmu.2022.869057

**Published:** 2022-04-12

**Authors:** David Michael Lewinsohn, Deborah Anne Lewinsohn

**Affiliations:** ^1^ Department of Medicine, Oregon Health and Science University, Portland, OR, United States; ^2^ Pulmonary and Critical Care Medicine, Portland VA Medical Center, Portland, OR, United States; ^3^ Department of Pediatrics, Oregon Health and Science University, Portland, OR, United States

**Keywords:** CD4/CD8 lymphocytes + +, MTB, cytolytic, intracellular infection, granuloma

## Abstract

For most vaccination studies, the assessment of vaccine-induced CD4^+^ and CD8^+^ T cells has relied upon the measurement of antigen-specific polyfunctional cells, typically using recombinant antigen or peptide pools. However, this approach leaves open the question as to whether or not these cells are responsive to the Mtb-infected cell within the context of Mtb infection and hence leaves open the possibility that a key parameter of vaccine immunogenicity may be overlooked. In this review, we discuss the case that these measurements almost certainly over-estimate the capacity of both CD4^+^ and CD8^+^ T cells to recognize the Mtb-infected cell.

## Introduction

Despite widespread use of the Bacille Calmette-Guérin (BCG) vaccine, tuberculosis (TB) remains a major cause of morbidity and mortality worldwide (1). While BCG is partially effective against severe TB in young children, BCG has little to no impact on preventing *Mycobacteria tuberculosis* (Mtb) infection or TB in older children or adults (2). Therefore, a more effective TB vaccine is urgently needed. Yet, development of improved TB vaccines requires overcoming multiple challenges. One of the most important challenges is the need to define robust correlates of protection, the absence of which hampers both pre-clinical research and human clinical trials. As is relevant to human clinical trials, most otherwise healthy adults who develop TB do so within 1-2 years following infection, as indicated by the conversion of a tuberculin skin test (TST) or interferon-γ release assay (IGRA) from negative to positive (3, 4). In addition, as compared to other infectious pathogens, diagnosis of TB disease is relatively resource-intensive. These attributes of TB require that human TB vaccine clinical trials are relatively long and resource-intensive, as compared to vaccine trials for other human pathogens. Therefore, development of robust correlates of protection have the potential to greatly expedite TB vaccine research, as this would allow for more timely evaluation of vaccine candidates. Most certainly, a panel of correlates of protection will be required that best reflect the efficacy of TB vaccine candidates utilizing different vaccine platforms (e.g. viral vectors, recombinant protein, attenuated mycobacteria), in various populations (e.g. young children, adults, people living with HIV) and along the spectrum of infection to disease (e.g. prevention of infection, prevention of disease).

Correlates of protection are defined as laboratory parameters, which are associated with protection from the occurrence of a clinical endpoint after well-controlled trials (5). These correlates can be further defined as laboratory parameters, which are causally responsible for protection (mechanistic correlates of protection) versus those that are significantly correlated with, though not causally responsible for protection (non-mechanistic correlates of protection) (6). The TB vaccine field has focused primarily on evaluating the phenotype and function of T cells that mediate successful host defense as candidate mechanistic correlates of protection. Herein, we examine the role of recognition of the Mtb-infected cell in successful host defense. Furthermore, we propose that a clinically refined assay to determine if vaccine-induced T cells recognize the Mtb-infected cell may represent a robust mechanistic correlate of protection that could expedite TB vaccine development.

## How Might T Cells Control Mtb?

T cells are thought to play a critical role in the containment of infection with Mtb. Specifically, both CD4^+^ and CD8^+^ T cells have been associated with the control of Mtb in both mouse and non-human primate (NHP) models. Phenotypically, the production of cytokines such as IFN-γ, TNF-α, and IL-17, growth factors such as GM-CSF, as well as components of the cytolytic machinery have all been associated with the control of Mtb (7, 8).

However, in humans, the precise mechanisms by which T cells can control Mtb remain poorly defined. For example, a central hypothesis of intracellular control of Mtb rests on the concept that myeloid cells can contain intracellular growth. Specifically, it is commonly stated that proinflammatory cytokines, such as TNF-α or IFN-γ, can induce the production of NOS2, which in turn leads to the production of NO, and subsequent mycobacterial control (9). While NOS2 is clearly required for the control of Mtb in mice, its role in humans is much less clear. In human upper airway epithelial cells, NOS2 is readily measured, is associated with NO that can be measured in exhaled gas, and has been used as a marker for airway inflammation (10). In addition, NO has been observed in macrophages contained in the human granuloma (11, 12). However, direct demonstration of the presence of NOS2 or the production of NO following cytokine stimulation of human monocytes or macrophages has not been observed in the literature. Therefore, while NO is present in the human lung within the context of Mtb infection, we speculate that this reflects a coordinated multi-cellular response, inclusive of T cells.

In this regard, T cells could facilitate mycobacterial control either directly or indirectly. Implicit in a direct approach is a physical interaction of the T cell and the Mtb-infected cell. Here, introduction of components of the cytolytic granule can induce autophagy or play a direct role in mycobacterial control though the introduction of molecules such as granulysin or IL-26 (13, 14). Alternately, T cells could inhibit mycobacterial growth indirectly *via* the release of cytokines and/or chemokines which in turn could enable myeloid cell control of Mtb, or direct the trafficking of additional effectors. In thinking about the mechanisms by which T cells might contribute to mycobacterial control, the question of whether or not the T cell can “sense” the Mtb-infected cell becomes central. Detection of the Mtb-infected cell rests upon the presentation of Mtb antigens by MHC-I or MHC-II molecules to CD8^+^ or CD4^+^ T cells, respectively. As Mtb is an intracellular bacteria, its detection poses different challenges to the immune system than extracellular bacteria. For the most part, Mtb resides in an arrested late endosome. This compartment shares many features with the canonical MHC-II processing compartment, the MIIC, whose function is to process extracellular antigens. As will be discussed more below, this makes it difficult for CD4^+^ T cells to distinguish the Mtb-infected cell from an uninfected cell that has sampled extracellular Mtb proteins. On the other hand, the MHC-I processing pathway is designed to sample the intracellular environment, hence the CD8^+^ T cell is poised to distinguish infected from uninfected cells. At present, standard approaches to measure T cell prevalence and phenotype typically rely on stimulation with recombinant protein antigens or peptide pools, or staining with tetramers. As will be described below, these approaches almost certainly overestimate the ability of these cells, particularly those induced by vaccination, to actually recognize the infected cell.

## CD4^+^ T Cells: Hide and Seek

While Mtb can escape the phagosome, and hence have access to the cytosol (15), Mtb is primarily thought to reside in an arrested endosomal compartment with features similar to a late endosome (16). As the processing and presentation of antigens *via* the MHC-II pathway are dependent on the late endosome (17), it is possible that a myeloid antigen presenting cell could sample antigens that are either derived intracellularly, or sampled from the extracellular environment. However, there is emerging evidence that CD4^+^ T cells may be relatively impaired in their ability to discern cells containing Mtb. In a set of seminal experiments, Wolf et al., found that the initiation of CD4^+^ T cell responses did not occur in mice until Mtb-infected DC had migrated to the draining LN (18). Among other things, these data suggested that cognate interactions, that is those requiring the direct presentation of antigen *via* MHC-II, to CD4^+^ T cells *via* TCR engagement, are essential in priming the CD4^+^ T cell response. By using a mixed chimera approach, Srivastava et al., could demonstrate that control of mycobacteria growth *in vivo* was dependent on these cognate interactions (19). By the same token, it is becoming increasingly clear that these interactions occur less commonly, or less robustly, in the granuloma than might be expected. For example, Egen et al., used intravital imaging to examine the behavior of T cells within the granuloma (20). They found, that in contrast to exogenously added antigen, only small fractions of mycobacteria-specific T cells showed antigen-induced migration and arrest within granulomas. This in turn, was associated with only low-level secretion of cytokines. Similarly, Gern et al., found that IFN-γ production by T cells in the lung was impaired, and that this was associated with a TGF-β signature in granulomas in both mice and non-human primates (21). Finally, Billeskov et al., found that CD4 epitopes for the Mtb antigen 10.4 varied based on the route of either mycobacterial challenge or immunization (22).

These data, then, would argue that Mtb has evolved mechanisms to deflect cognate CD4^+^ T cell interactions. For example, Srivastava et al. have found that infection with Mtb enhances the vesicular export of antigens, and have suggested that this could be a mechanism to divert T cells away from the infected cell (19). It has also been observed that lipoproteins from Mtb can lead to downregulation of MHC-II, and hence diminished T cell recognition (23). At present, the mechanisms by which Mtb can avoid detection from CD4^+^ T cells remains incompletely understood, but it is clear that our current approaches to enumerating Mtb-specific CD4^+^ T cells will overestimate their capacity to respond to the infected cell.

## CD8^+^ T Cells: Looking in All the Wrong Places

As mentioned above, it has been argued that control of Mtb requires a cognate MHC-II, CD4^+^ T cell interaction. However, it is not clear how CD4^+^ T cells can discern the infected cell from one that has sampled antigen derived from the environment. CD8^+^ T cells are uniquely capable of discerning Mtb-infected cells, particularly those that are HLA-II negative. Human Mtb-specific CD8^+^ T cells are further distinguished by both their preferential recognition of heavily infected cells and restriction by HLA-B (24, 25). It is increasingly evident that CD8^+^ T cells have an important and complex role in Mtb containment and immunity (26) particularly in the long-term progression of mycobacterial growth that has been demonstrated in the mouse and non-human primate models (26).

CD8^+^ T cells primarily sample intracellular antigen, which is dependent upon a cognate interaction with the infected cell. It has been hypothesized, that this in turn is dependent upon escape of Mtb from the phagosome into the cytosol mediated by proteins encoded by the RD1 region of Mtb (27). However, RD1-deficient Mtb are efficiently recognized by human CD8^+^ T cells (28) and it has been demonstrated that the Mtb phagosome itself can contribute to MHC-I processing and presentation (29). In addition to CD8^+^ T cell recognition of infected cells, these cells may also recognize Mtb antigens derived from apoptotic cells taken up by DC, *via* a mechanism broadly termed cross-presentation (30). DC play a central role in the initiation or “priming” of the Mtb-specific CD8^+^ T cell response. Here, this could occur either *via* cognate interactions with Mtb-infected DC or possibly *via* cross-presentation. Once primed, CD8^+^ T cells can directly sense Mtb-infected cells (25, 31, 32), and this recognition may depend on the magnitude of the intracellular infection (24). However, how these CD8^+^ T cells recognize infected cells *in vivo* is not well understood and will depend upon additional animal studies.

For CD8^+^ T cell recognition to occur, antigens must be expressed intracellularly by Mtb. At present, much of this work has focused either on identifying the antigens recognized by T cells derived from the peripheral circulation, or *via* the infection of myeloid cells such as macrophages and DC. As a result, our understanding of antigens presented in the context of non-myeloid cells such as epithelial (33) and endothelial cells (34), and specifically in the lung and granuloma remains incomplete (35, 36). Regardless of the mechanisms by which this processing and presentation occurs, it is increasingly clear that not all antigens expressed by Mtb can be recognized by CD8^+^ T cells. The antigen 10.4, first described by Skjot et al., is an excellent case in point (37). In the context of infection with Mtb, this antigen is robustly recognized by both mouse and human CD4^+^ and CD8^+^ T cells (38). Furthermore, it is considered a leading candidate for inclusion in vaccine candidates (39). In the Mtb-infected C57BL/6 mouse, the 10.4^3-11^/10.4^4-11^ epitope is immunodominant (32, 38), and Woodward et al., found that adoptive transfer of immune CD8^+^ T cells isolated from wild-type, but not perforin-deficient C57BL/6 mice, reduced the bacillary burden in recipient mice (40). Surprisingly, then, Yang et al (41), went on to demonstrate that the Mtb-infected APC were not able to activate 10.4^4-11^ specific CD8^+^ T cells, suggesting that this epitope was not functionally displayed by these cells (41). At a minimum, this finding would suggest that the antigens presented during T cell priming could be distinct from those displayed by Mtb-infected cells. It is also possible that non-myeloid cells could express this epitope. The authors argue that the expansion of cells unable to recognize the Mtb-infected cell could be one strategy for immune evasion. In this regard, Sutiwisesak et al. were able to challenge mice with a clinical strain displaying an altered 10.4 epitope, which in turn altered the pattern of immunodominance of the CD8^+^ T cell response to 10.4. These cells, in turn, were better able to recognize the Mtb-infected cell (42).

In humans, we have typically found that Mtb-specific CD8^+^ T cells isolated from individuals naturally infected with Mtb are capable of recognizing the Mtb-infected cell (25, 31, 43). However, there is evidence to support the hypothesis that vaccination may elicit T cells recognizing epitopes that are not displayed by the Mtb-infected cell. In this regard, we have previously published the results of a phase I double-blind, randomized placebo-controlled trial of vaccination with AERAS-402 following BCG. AERAS-402 is a recombinant replication-deficient serotype 35 adenovirus expressing a fusion protein of Mtb antigens 85A, 85B and TB10.4. Analysis of the vaccine-induced immune response revealed strong antigen-specific polyfunctional CD4^+^ and CD8^+^ T cell responses. However, analysis of the vaccine-induced CD8^+^ T cells revealed that in many instances these cells did not recognize the Mtb-infected cell (44).

## Discussion

For most vaccination studies, the assessment of vaccine-induced CD4^+^ and CD8^+^ T cells has relied upon the measurement of antigen-specific polyfunctional cells, typically using recombinant antigen or peptide pools. However, this approach leaves open the question as to whether or not these cells are responsive to the Mtb-infected cell within the context of Mtb infection. Hence this leaves open the possibility that a key parameter of vaccine immunogenicity may be overlooked.

With regard to CD4^+^ T cells, it would seem that the CD4^+^ T cell response is blunted *in vivo* in the infected tissues. At present, it is not feasible to assess this diminished tissue T cell responsiveness using PBMC. However, the assessment of the quality of the CD4^+^ T cell response within the context of the lung and/or granuloma in animal models could serve as an adjunct in the development of a pre-clinical portfolio for promising vaccine candidates.

With regard to CD8^+^ T cells, our characterization of those epitopes that were elicited by vaccination and then discerning those that were presented by the Mtb-infected cell required isolation and detailed characterization of T cell clones (44). Hence this fits into the paradigm of a dedicated, Phase I experimental medicine study. True correlates of protective immunity within the context of vaccination will necessarily be predicated on the detailed analysis of vaccine-induced protection, as was seen in the M72/AS01_E_ Vaccine trial (45). Here, flow cytometric methods to directly assess the ability of vaccine-induced T cells to recognize the Mtb-infected cell should be prioritized, and clinically validated. In this regard Patankar et al., have used ELISPOT to compare relative frequencies of CD8^+^ T cells responding to the Mtb-infected versus peptide-loaded APC among polyclonal populations (46). Furthermore, it would be ideal to directly measure cognate interactions between effector T cells and Mtb-infected cells. One example of such an assay is one that reflects cognate induction of apoptosis by CD8^+^ T cells (47). Another example would be an assay that detects the transfer of components of the cytolytic granule, such as granzyme B, from the CD8^+^ T cells to Mtb-infected cells (48). Most recently Liu et al., described a method utilizing cell-surface enzymatic fucosyl-biotinylation from CD8^+^ T cells to the target APC to define cognate interactions (49). For these approaches to be successful, it will be necessary to identify the cells recognized by CD8^+^ T cells, to distinguish infected and uninfected target cells and ideally to assess the viability of these microbes. In this regard Bryson et al. have engineered a strain of Mtb that express a constitutive fluor in conjunction with an inducible fluor that tags intracellular Mtb and allows for the determination of viability (50).

## Author Contributions

DML and DAL contributed equally to the review with regard to the creation of the outline, research, and writing. All authors contributed to the article and approved the submitted version.

## Funding

Grant support I01 BX000533/BX/BLRD VA/United States R01 AI134790/AI/NIAID NIH HHS/United States R01 AI147954/AI/NIAID NIH HHS/United States.

## Conflict of Interest

The authors declare that the research was conducted in the absence of any commercial or financial relationships that could be construed as a potential conflict of interest.

## Publisher’s Note

All claims expressed in this article are solely those of the authors and do not necessarily represent those of their affiliated organizations, or those of the publisher, the editors and the reviewers. Any product that may be evaluated in this article, or claim that may be made by its manufacturer, is not guaranteed or endorsed by the publisher.
